# Machine learning to identify phenotypic clusters of patients with atrial fibrillation

**DOI:** 10.1016/j.hroo.2024.12.001

**Published:** 2024-12-12

**Authors:** Hani Essa, Sandra Ortega-Martorell, Ivan Olier, Gregory Y.H. Lip

**Affiliations:** 1 Liverpool Centre for Cardiovascular Science at University of Liverpool, Liverpool Heart and Chest Hospital, Liverpool John Moores University, Liverpool, United Kingdom; 2 Data Science Research Centre, Liverpool John Moores University, Liverpool, United Kingdom; 3 Danish Center for Health Services Research, Department of Clinical Medicine, Aalborg University, Aalborg, Denmark; 4 Department of Cardiology, Lipidology and Internal Medicine, Medical University of Bialystok, Bialystok, Poland

Atrial fibrillation (AF) is the most common cardiac arrhythmia worldwide and is associated with significant morbidity and mortality, including stroke, heart failure, dementia, and hospitalizations.^1^ As a result, better efforts to identify patients at greatest risk, who would benefit most from appropriate management, are needed.

Oral anticoagulation can be used to ameliorate the risk of stroke in AF, but the management of AF is more than just oral anticoagulation, given the recognized residual risks of major adverse events despite anticoagulation.^2^ Indeed, AF is not a homogeneous single diagnosis, and over recent years, phenotypes of “clinical complexity” associated with AF have been identified, with implications for prognosis and management.^3^

The current management of AF has moved toward a more holistic or integrated care approach, initially proposed as the ABC (Atrial fibrillation Better Care) pathway.^4^ The ABC pathway is supported by trial and real-world evidence,^5^ and variants of the “ABC” acronym have been used in U.S. guidelines (ie, SOS [Stroke, Other Comorbidities, Rate or Rhythm control])^6^ and 2024 European guidelines (as CARE [ie, Comorbidities, Avoid stroke, Rate or rhythm control, Evaluation]).^7^

Phenotypic clusters of AF patients identified by hierarchical cluster analysis show improved outcomes with ABC pathway adherence but to a varying degree depending on their phenotype.^3^ Adherence to the ABC pathway has been associated with a significant reduction in all-cause mortality, cardiovascular mortality, stroke, and bleeding.^8^

Turning to stroke risk, the more common and well-validated risk factors have been utilized to formulate stroke risk stratification schemes, of which the one most used is the CHA_2_DS_2_-VASc (congestive heart failure, hypertension, age ≥75 years, diabetes mellitus, prior stroke or transient ischemic attack or thromboembolism, vascular disease, age 65–74 years, sex category) score.^9^ While clinically useful to identify patients who may benefit from anticoagulation, this is an oversimplification of a much more complex and dynamic scenario, and hence only demonstrates a modest predictive performance of stroke risk. There is a clinically apparent need for better risk-stratification strategies to identify patients who may benefit from anticoagulation.

Beyond hierarchical cluster analysis, other approaches such as latent class analysis have been used to phenotype patients with AF.^10^ Nevertheless, artificial intelligence (AI) and machine learning (ML), a subset of AI, signal the emergence of tools that can help us leverage large data sets to identify clinically significant patterns that may not be easily identified by conventional methods, and this is demonstrated in dramatic growth in the numbers of publications in this field over the last few years.^11^

In this issue of *Heart Rhythm O*^*2*^, Hsu and colleagues^12^ used a statistical approach to identify distinct prognostic phenotypic clusters in a Taiwanese population of 5002 patients with AF. In this analysis, the authors perform an unsupervised hierarchical cluster analysis based on the components of the CHA_2_DS_2_-VASc score, identifying 4 distinct clusters: cluster I included 1918 diabetic patients with heart failure with preserved ejection fraction, and chronic kidney disease; cluster II comprised 1006 older patients with low body mass index and pulmonary hypertension; cluster III consisted of 1731 patients with metabolic syndrome and atherosclerotic disease; and cluster IV included 347 patients with left heart dysfunction, including reduced ejection fraction.

The main outcomes measured across all clusters were the risk of ischemic stroke, heart failure hospitalization, cardiovascular death, and all-cause mortality. First, Hsu and colleagues found significant differences in the risk of ischemic stroke independent of CHA_2_DS_2_-VASc score between clusters, with cluster IV demonstrating the lowest risk. Second, cluster II was associated with the highest risk of heart failure hospitalization, cardiac death, and all-cause mortality. Finally, the data-driven algorithm identified heterogeneous risk profiles across different clusters, each associated with a varying risk of cardiovascular events. The study by Hsu and colleagues identified distinct phenotypes that demonstrated a differential risk of stroke, independent of their CHA_2_DS_2_-VASc score. The authors further benefitted from a large sample size and a long duration of follow-up. Furthermore, these results were externally validated on a separate dataset, lending creditability and demonstrating reproducibility of the findings.

Nonetheless, several limitations must be considered when interpreting these results. First, this is a retrospective study in a predominantly homogeneous East Asian population and is therefore open to confounding factors that may explain these findings. Second, as a study dependent on data extraction from an administrative database (without a review of individual patient charts), there is a significant risk that data are subject to coding errors, which can alter results. Last, even though their work is supported by external validation on a Taiwanese dataset, its applicability to non-Asian populations is uncertain, especially given the reported racial differences in AF-related outcomes such as stroke and bleeding.^13^^,^^14^

The work conducted by Hsu and colleagues is timely and contributes to the expanding role of data-driven approaches in the management of AF.^11^ Moving forward, phenotyping clinically complex AF patients can deploy more ML sophisticated approaches, such as generative topographic mapping, as recently published by our group.^15^ AI/ML has the potential to be able to provide a continuous “real-time” assessment of individual risk in AF outperforming traditional stroke risk stratification schemes.^16^ This is augmented by the growth in methodologies such as digital twins, currently applied in extensive research programs to improve diagnosis, risk prediction, peristroke management, and poststroke rehabilitation.^17^^,^^18^

However, risk factors and comorbidities in AF patients are not static, but rather are dynamic in nature, and the arrhythmia per se is also dynamic, changing in patterns over time.^19^^,^^20^ Furthermore, we have adherence to the ABC pathway that is also dynamic, and adherence/nonadherence over follow-up can impact outcomes.^21^

**Figure 1:**
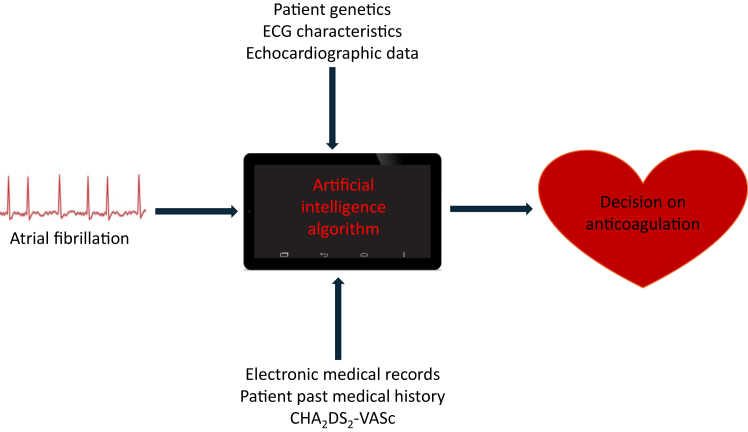
The potential role of artificial intelligence and machine learning in decision making for anticoagulation. CHA_2_DS_2_-VASc = congestive heart failure, hypertension, age ≥75 years, diabetes mellitus, prior stroke or transient ischemic attack or thromboembolism, vascular disease, age 65–74 years, sex category; ECG = electrocardiography.

Novel ML approaches could help identify phenotypic clusters of AF patients, who have a high risk of ischemic stroke, despite being deemed low risk by traditional risk scores such as CHA_2_DS_2_-VASc and randomizing these patients to ABC pathway–based management vs conventional treatment. The ability to identify these high-risk individuals who would otherwise be missed could allow a tailored approach to decision making and improve overall patient care. Ultimately, we may utilize AI/ML to create an algorithm that incorporates conventional patient data used in normal risk stratification schemes combined with nonconventional and dynamic data for the identification of which patients may benefit from anticoagulation (Figure 1).

In conclusion, the growth in AI/ML yields promising results for the identification of high-risk patients who may otherwise be missed via conventional stroke stratification schemes. Integrating AI/ML into the diagnostic and treatment processes for AF has the potential to mitigate current limitations and optimize care. The “rise of the machines” is clearly evident in healthcare.
